# A Case of Visual Hallucination With Frontal Lobe Infarction in a Patient With Giant Cell Arteritis

**DOI:** 10.7759/cureus.41659

**Published:** 2023-07-10

**Authors:** Mai Shimura, Hirohisa Fujikawa, Masei Suda, Kiyoharu Muranaka, Masahiro Minoda

**Affiliations:** 1 Department of Internal Medicine, Suwa Central Hospital, Nagano, JPN; 2 Department of Medical Oncology, National Cancer Center Hospital East, Chiba, JPN; 3 Center for General Medicine Education, School of Medicine, Keio University, Tokyo, JPN

**Keywords:** frontal lobe, magnetic resonance imaging, visual hallucination, ischemic stroke, giant cell arteritis (gca)

## Abstract

Giant cell arteritis (GCA) can produce a variety of visual symptoms. Among these, visual hallucinations are rare and are usually accompanied by visual loss. We encountered a 79-year-old female with GCA who presented with visual hallucinations without visual loss. Magnetic resonance imaging (MRI) of the head revealed a stroke in the right frontal lobe, probably caused by GCA, resulting in visual hallucinations. Visual hallucinations are not well recognized by clinicians as a presentation of GCA. However, as shown in the present case, visual hallucinations are an important symptom because they are suggestive of cerebral ischemia or visual loss.

## Introduction

Giant cell arteritis (GCA) is the most common form of systemic vasculitis affecting medium to large vessels with a marked female predominance [1]. According to a population-based study, 40-60% of patients with GCA have polymyalgia rheumatica (PMR), and GCA is present in 16-21% of patients with PMR [1]. This incidence rate increases after 50 years of age [1]. With an aging population, the occurrence of GCA is expected to increase in the near future. De Smit et al. estimated that by 2050, over three million people will be diagnosed with GCA [2].

GCA can cause a variety of ocular manifestations. Visual hallucinations are a rare but serious complication that can reduce the quality of life. Visual hallucinations can occur in the context of psychiatric disorders, but they can also occur in patients who have brain lesions or are using certain medications [3,4]. According to previous reports, visual hallucinations in patients with GCA are usually associated with visual loss [3-5]. Herein, we report a rare case of GCA with visual hallucinations and no visual loss, probably caused by a frontal lobe infarction due to GCA.

## Case presentation

**Figure 1:**
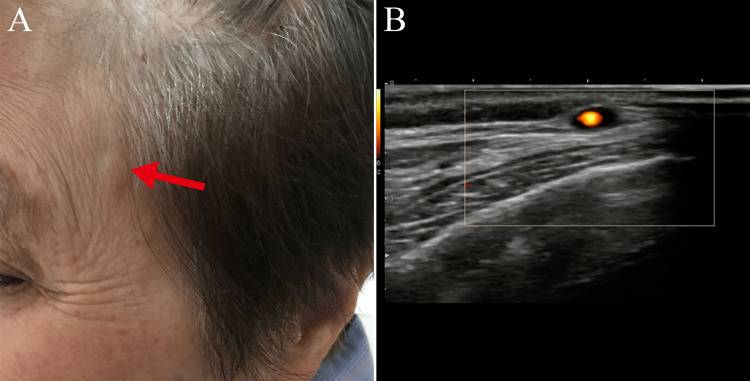
Patient photograph and ultrasound image on admission. (A) Left superficial temporal artery is tortuous and prominent (red arrow). (B) Left superficial temporal artery ultrasonography shows wall thickening (dark halo sign).

**Figure 2:**
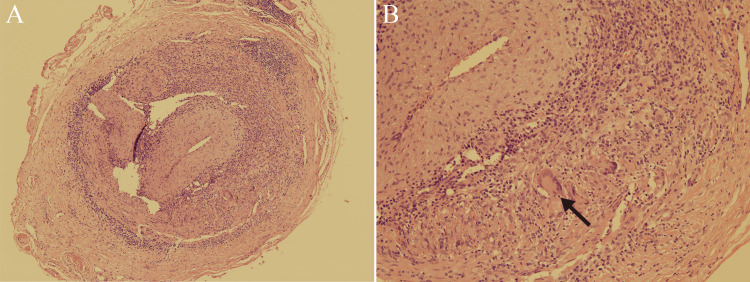
Pathological examination of the left superficial temporal artery. (A) There is marked lymphocyte, plasma cell, and histiocyte infiltration in all layers of the vessel. The intima of the vessel is thickened, and the lumen of the vessel is prominently narrowed (hematoxylin and eosin stain, original magnification x40). (B) There are some giant cells (arrow) in the arterial media (hematoxylin and eosin stain, original magnification x100).

A 79-year-old female with no cognitive impairment presented with a three-day history of a tinkling sensation in the eyes (“like light reflecting off a lake”). She had developed myalgia and a bilateral temporal headache three weeks prior. On examination, bilateral superficial temporal arteries were tortuous and prominent (Figure 1A). Laboratory investigations showed a C-reactive protein level of 50.7 (normal range 0-3) mg/L and erythrocyte sedimentation rate of 110 (normal range < 30) mm/hour. Musculoskeletal ultrasound of the shoulder revealed subacromial bursitis. Bilateral superficial temporal artery ultrasound showed non-compressible arteries (compression sign) and wall thickening (dark halo sign; Figure 1B). Left temporal artery biopsy revealed marked lymphocyte, plasma cell, and histiocyte infiltration in all layers of the vessel, and some giant cells in the arterial media (Figure 2A, 2B). We made the diagnosis of GCA with PMR and initiated 1 mg/kg/day of methylprednisolone.

**Figure 3:**
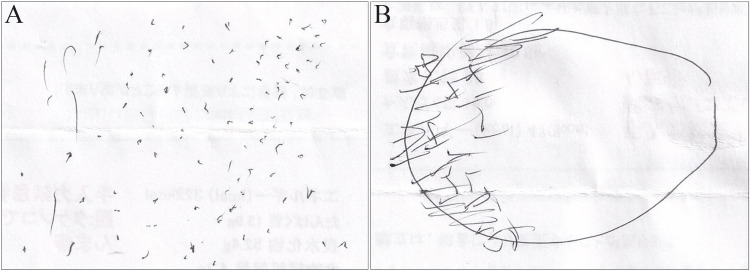
The patient’s own sketches of the content of visual hallucinations ((A) sesame, (B) shiny lake surface).

A few days after initiation of treatment, the headache and myalgia abated, and inflammatory markers decreased. Conversely, the ocular symptoms gradually worsened. Ophthalmological assessment revealed normal visual acuity in both eyes, with no indication of ischemic eye disease. In the following days, the patient reported seeing various things (Figure 3A, 3B): “black straw,” “green bamboo grove,” “sesame,” and “shiny lake surface.” Each visual hallucination lasted several hours. She was consistently alert and oriented and was aware that she was having visual hallucinations. Her mental status was normal, and she presented with no attention, language, motor, or sensory deficits. Ophthalmological assessments were repeated, all of which were normal. The patient’s progress was good, despite visual symptoms. After a week of admission, her medication was changed to 1 mg/kg/day of oral prednisolone. On the ninth day of hospitalization, she was discharged.

**Figure 4:**
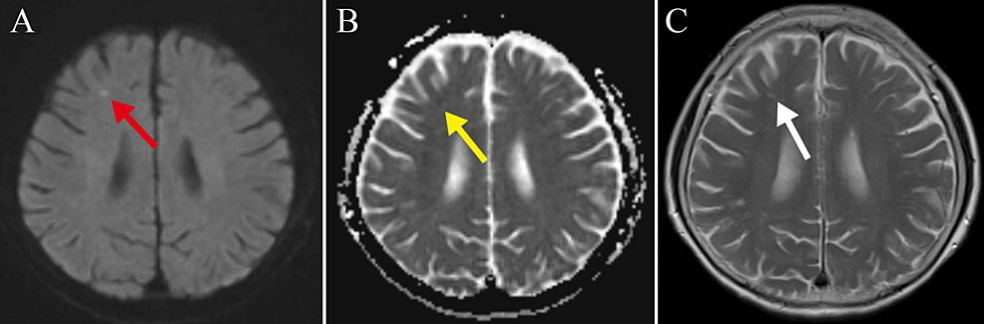
Cranial magnetic resonance imaging images two weeks after discharge. (A) Diffusion-weighted imaging shows a high signal in the right frontal lobe (red arrow). (B) Apparent diffusion coefficient map shows a low signal in the right frontal lobe (yellow arrow). (C) T2-weighted imaging shows a high signal in the right frontal lobe (white arrow).

Two weeks post-discharge, she had a follow-up outpatient visit with no complaints of myalgia, headache, and ocular symptoms. However, on the magnetic resonance imaging (MRI), we detected a lesion in the right frontal lobe showing hyperintensity on diffusion-weighted images, hypointensity on apparent diffusion coefficient maps, and hyperintensity on T2-weighted images (Figure 4A-4C), findings that are consistent with a subacute to acute stroke. Holter electrocardiography, echocardiography, and carotid ultrasonography illuminated no cause of the stroke. Based on the above findings, GCA was conjectured as the cause of the cerebral infarction. Prednisolone was tapered, and she has had no symptom recurrence to date.

## Discussion

Patients with GCA exhibit various manifestations, including headache, visual symptoms, musculoskeletal involvement, and nonspecific symptoms (e.g., fever and fatigue). The visual symptoms are notable because acute ocular manifestations of GCA require urgent evaluation and treatment to avoid permanent vision loss. The incidence of visual symptoms in GCA is common, ranging from 12% to 70% [6]. Permanent visual loss is a much-feared consequence of GCA and occurs in up to 30% of patients with GCA [7]. Arteritic anterior ischemic optic neuropathy, central retinal artery occlusion, cilioretinal artery occlusion, and occipital lobe infarction are known causes of permanent vision loss, whereas diplopia in GCA is representatively transient and can result from ischemia in any region of the oculomotor system (i.e., brainstem, ocular motor nerves, and extrinsic ocular muscles) [6-8].

Visual hallucinations are a less common ocular presentation of GCA and are poorly recognized by clinicians. Five articles on visual hallucinations in patients with GCA have been published (Table 1) [3-5,9,10]. Most of these patients experienced visual hallucinations alongside visual loss, the “Charles Bonnet syndrome,” a result of the “release” of the primary visual cortex due to visual sensory deafferentation [11].

**Table 1:** Clinical characteristics of patients with giant cell arteritis and visual hallucination. F: female; M: male; NR: not reported

Author (year) [Ref]	Number of patients	Age	Sex	Permanent visual loss, N (%)	Content of visual hallucinations
Hart (1967) [9]	2	73, 79	F	2 (100)	Blue and white dishes, flapping brightly colored curtains, etc.
Sonnenblick (1995) [10]	1	87	F	1 (100)	NR
Nesher (2001) [3]	4	71-82	3 F, 1 M	4 (100)	Cats, colorful rays, mice, etc.
Liozon (2002) [5]	14	NR	NR	3 (21)	NR
Razavi (2004) [4]	1	79	F	1 (100)	Birds, flashing lights, flying bugs, etc.
Present case	1	79	F	0 (0)	Black straw, shiny lake surface, green bamboo grove, the shadow of a person

Conversely, visual hallucinations without visual loss are rare in patients with GCA. In this case, MRI revealed a subacute to acute frontal lobe infarction, which is a potential cause of visual hallucinations. Historically, presentations of Parkinson’s disease or Lewy body dementia with visual hallucinations had a greater frontal gray matter atrophy than those with no visual hallucinations [12]. Parkinson’s patients with visual hallucinations showed reduced performance on tasks that explore executive functioning, suggesting relative frontal dysfunction, as compared with non-hallucinators [13]. Considering this information, visual hallucination in this case was probably caused by the frontal lobe infarction. Liozon et al. reported GCA patients with visual hallucinations and without visual impairment [5], although no head MRI was performed, and their pathophysiology was unclear. This case report identifies the pathogenesis of visual hallucinations without visual impairment in a patient with GCA.

## Conclusions

Here, we report a case of GCA with visual hallucinations and without visual loss. Visual hallucinations are under-recognized as presentations of GCA. As visual hallucinations can signify brain ischemia or visual loss, physicians should be aware of these symptoms and consider brain MRI testing and ophthalmological evaluation.
